# Pulmonary artery closure in combination with patch technique for treating congenital heart disease combined with large patent ductus arteriosus: A clinical study of 9 cases

**DOI:** 10.12669/pjms.323.9872

**Published:** 2016

**Authors:** Bing Wen, Junya Yang, Huiruo Liu, Zhouyang Jiao, Wenzeng Zhao

**Affiliations:** 1 Bing Wen, Department of Cardiovascular Surgery, the First Affiliated Hospital of Zhengzhou University, Zhengzhou 450052, China; 2 Junya Yang, Department of Dermatological, the Fifth Affiliated Hospital of Zhengzhou University, Zhengzhou 450052, China; 3 Huiruo Liu, Department of Cardiovascular Surgery, the First Affiliated Hospital of Zhengzhou University, Zhengzhou 450052, China; 4 Zhouyang Jiao, Department of Cardiovascular Surgery, the First Affiliated Hospital of Zhengzhou University, Zhengzhou 450052, China; 5 Wenzeng Zhao, Department of Cardiovascular Surgery, the First Affiliated Hospital of Zhengzhou University, Zhengzhou 450052, China

**Keywords:** Hybrid surgery, Occlusion, Patent ductus arteriosus

## Abstract

**Objective:**

To document clinical experience of treating congenital heart disease combined with large patent ductus arteriosus with pulmonary artery closure in combination with patch technique.

**Methods:**

Thirty-six patients (8 males and 28 females) who suffered from congenital heart disease and underwent hybrid surgery in the First Affiliated Hospital of Zhengzhou University from October 2010 to February 2014 were selected for this study. They aged 14 to 39 years and weighed 32.20 to 61.50 kg. Diameter of arterial duct was between 10 mm and 13 mm; 28 cases were tube type, 4 cases were funnel type and four cases were window type. All patients had moderate or severe pulmonary arterial hypertension; besides, there were 28 cases of ventricular septal defect, 16 cases of atrial septal defect, eight cases of aortic insufficiency, four cases of mitral stenosis and insufficiency and four cases of infectious endocarditis. Cardz Pulmonary Bypass (CPB) was established after chest was opened along the middle line. With the help of Transesophageal echocardiography, large patent ductus arteriosus was blocked off through pulmonary artery. Pulmonary artery was cut apart after blocking of heart. Large patent ductus arteriosus on the side of pulmonary artery was strengthened with autologous pericardial patch.

**Results:**

Of 36 patients, 32 patients had patent ductus arteriosus closure device and four patients had atrial septal defect closure device. Pulmonary arteries of 36 cases were all successfully closed. Systolic pressure declined after closure ((54.86±19.23) mmHg vs (96.05±23.07) mmHg, p<0.05); average pulmonary arterial pressure also declined after closure ((39.15±14.83) mmHg vs (72.88±15.76) mmHg, p<0.05). The patients were followed up for one to fifty one months (average 11.5 months). Compared to before surgery, left atrial diameter, left ventricular diameter and pulmonary artery diameter all narrowed after surgery. Besides, clinical symptoms were relieved and cardiac function of the patients also improved.

**Conclusion:**

Hybrid surgery is feasible and safe in treating patients with large patent ductus arteriosus and congenital heart disease, which decreases surgical problems, shortens surgical time and lowers the incidence of complications.

## INTRODUCTION

Patent ductus arteriosus, a commonly seen congenital heart disease, accounts for 12%~15% of congenital heart disease; 10% of cases of patent ductus arteriosus also combine other heart malformations; large patent ductus arteriosus (diameter larger than 10 mm) is more likely to induce severe pulmonary arterial hypertension.^1^,^2^ Nine patients included in this study suffered from large patent ductus arteriosus as well as multiple heart malformations and moderate or severe pulmonary arterial hypertension. Currently, the key of treatment for patent ductus arteriosus in combination with severe pulmonary arterial hypertension lies on the confirmation of reversibility of pulmonary arterial hypertension.^3^,^4^ Surgical operation is usually of high risks, especially when the reversibility of pulmonary arterial hypertension is difficult to be confirmed. Compared to surgical operation, interventional closure has been recognized in clinic for it can probe the properties of pulmonary arterial hypertension before the release of closure device.^5^-^7^

Conventionally after establishment of Cardio Pulmonary Bypass (CPB), patent ductus arteriosus is closed by patching and then the other malformations are processed.^8^ It is a challenge for cardiac surgeon to treat large patent ductus arteriosus in combination with moderate or severe pulmonary arterial hypertension using the above surgical method. That is because, high-pressure blood flow from main artery makes exposure difficult and severe outcomes such as perfusion lung and severe damage on blood components may be induced.^9^,^10^ To repair patent ductus arteriosus, hypothermia and flow perfusion and even suspension of circulation are usually adopted; however, the incidence of extracorporeal circulation associated complications becomes higher in such a condition. Our department started using hybrid surgery for treating large patent ductus arteriosus in combination with pulmonary arterial hypertension from September, 2010 and have achieved satisfactory clinical effects.

## METHODS

### Clinical data

Thirty-six cases including 8 males and 28 females were confirmed by transthoracic echocardiography before surgery. They aged from 14 to 39 years and weighed from 32.30 kg to 61.50 kg. Diameter of arterial duct was between 10 mm and 13 mm; 28 cases were tube type, four cases were funnel type and four cases were window type. The pattern of arterial duct shunt of all cases was left-to-right and they all developed moderate or severe pulmonary arterial hypertension (pulmonary arterial systolic pressure 59 mmHg~87 mmHg). Besides, there were 28 cases of ventricular septal defect, 16 cases of atrial septal defect, 8 cases of aortic insufficiency, four cases of mitral stenosis and insufficiency and four cases of infectious endocarditis. All patients gave informed consent. Patients who suffered from infectious endocarditis, neoplasm contained patent ductus arteriosus, other intracardiac malformations or other organ diseases were excluded. All patients had different degrees of palpitation and short of breath when overworked, which has lasted for one month to 20 years; there were 12 cases of hoarseness, 8 cases of chest pain, 6 cases of abdominal distension and edema of both lower limbs and one case of hemoptysis.

### Surgical protocol

**Fig.1:**
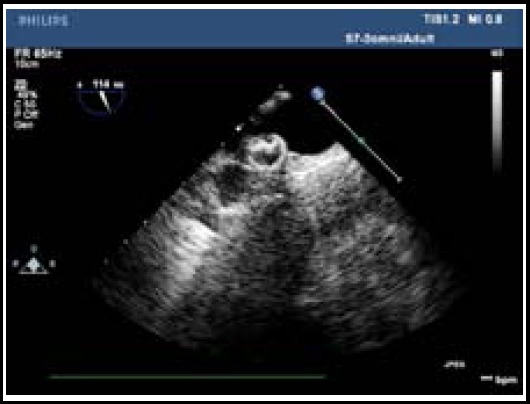
The release of closure device in descending aorta.

First general anesthesia and tracheal intubation were performed. After patients in supine position were processed with whole body heparinization, chest was open along the middle line and sternum was vertically split. Main pulmonary artery was exposed by suspending pericardium. Then the surface of pulmonary artery was sutured with purse string suture using 4-Oprolene suture. The selection of closure device was determined based on the diameter of patent arterial duct and the pattern of large patent ductus arteriosus measured using preoperative transthoracic echocardiography and intraoperative transesophageal echocardiogaphy. Close device selected was 4 mm~6 mm larger than the diameter of patent arterial duct and window-type patent dutus arteriosus with large diameter could be closed with atrial septal defect closure device. The products used were all produced by Shanghai Shanghai Shape Memory Alloy Material Co., Ltd. 14G trocar was used to puncture purse. After pin core was withdrawn, guiding wire was inserted through tubes. Under the guidance of transesophageal echocardiogaphy, the guiding wire entered descending aorta through arterial duct. Then the tube was withdrawn. The head end of sheathing canal was inserted into descending aorta along the guidance wire (Fig.1). After the expansion sheath and the guiding wire were withdrawn, 3-Oprolene suture penetrated the interval of metal wires inside stainless steel rivet of the closure device and then was drawn forth from the end of loading sheathing canal. Then the loading sheath canal which has been equipped with closure device and washed by heparin saline was connected with delivery sheath canal.

**Fig.2:**
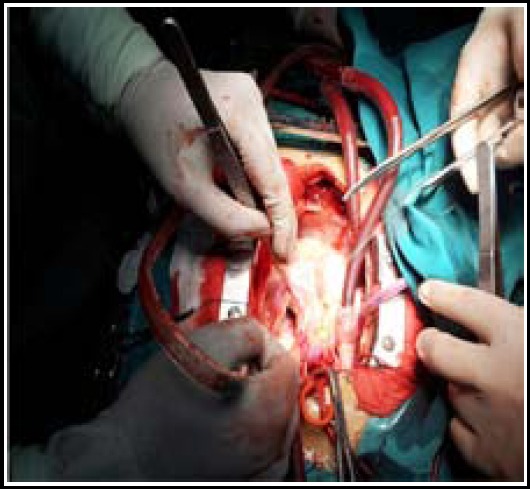
Strengthening with patching on pulmonary artery plane after closure.

**Fig.3:**
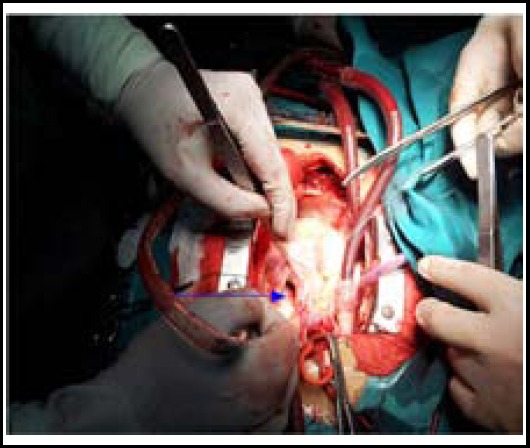
Pericardium is what the blue arrow points at.

Under the guidance of transesophageal echocardiogaphy, the closure device was pushed to the head end of sheath canal to release arterial plane and then retracted to close to aorta wall. The closure effect of four cases of window-type patent ductus arteriosus was poor; afterwards, 20 mm atrial septal defect was used. Transesophageal echocardiogaphy suggested the position of closure devices was appropriate and there was no residue shunt or residual shunt bundle was no more than 2 mm. After the delivery wire and sheath canal were removed, the preset safety wire was slightly pulled and fixed. CPB was established. After main artery was blocked, main pulmonary artery was cut apart to expose pulmonary artery plane. Bovine pericardium in proper size was selected and threaded with 3-Oprolene wire which was taken as safety wire. Then a knot was tied to firmly fix the pericardium (Fig.2). Patent ductus arteriosus was repaired by suturing bovine pericardium with 4-Oprolene wire (Fig.3). The other heart malformations were processed after patent ductus arteriosus was closed successfully.

### Observation indexes

Indexes of pulmonary arterial systolic pressure, mean pulmonary arterial pressure, aortic systolic pressure, mean aortic pressure, left atrium diameter (LAD), left ventricular end-diastolic diameter (LVEDD), pulmonary artery diameter (PAD) and left ventricular ejection fraction (LVEF) were observed before and after surgery.

### Follow up

Patients were followed up one day, one month, three months, six months and one year after surgery. One year later, electrocardiography and transesophageal echocardiogaphy were performed every other year.

### Statistical analysis

Data collected were statistically analyzed using SPSS19.0. Measurement data were expressed as mean±SD. Comparison of mean was performed using pair t test or repeated measures analysis of variance. p<0.05 suggested difference was statistically significant.

## RESULTS

### Curative effect

**Fig.4:**
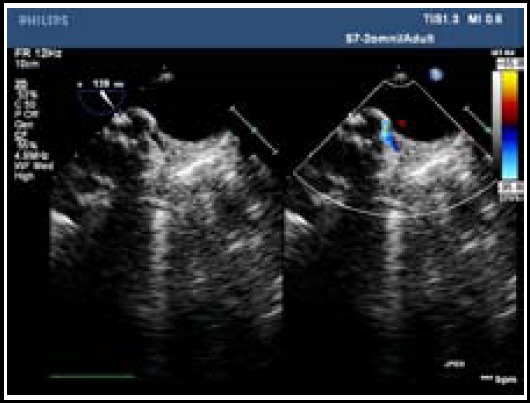
Blunt completely disappeared after the release of closure device.

Of 36 patients, 32 patients were equipped with patent ductus arteriosus closure device (14 mm~28 mm single rivet in column shape) and four patient was equipped with atrial septal defect closure device (20 mm). All cases were observed with well located closure device and without residual shunt by intraoperative transesophageal echocardiography and postoperative transthoracic echocardiography (Fig.4). Besides, one patient who developed infectious endocarditis was observed with pulmonary infection (fungal infection); the patient discharged from the hospital after 2-month treatment in department of respiration. The other patients discharged from the hospital within two weeks.

### Changes of arterial pressure

All patients had successful surgery and released closure device. Pulmonary arterial systolic pressure and mean pressure both declined after closure, while aortic systolic pressure and mean pressure both rose; differences were statistical significant (p<0.05) (Table-I).

**Table-I:** Changes of arterial pressure before and after closure (N=36).

	*Pulmonary arterial systolic pressure*	*Mean pulmonary arterial pressure*	*Aortic systolic pressure*	*Mean aortic pressure*
Before closure	96.05±23.07	72.88±15.76	137.27±18.76	87.94±10.93
After closure	54.86±19.23	39.15±14.83	142.72±19.43	97.51±9.72
t value	11.38	14.92	-2.28	-5.12
p value	0.000	0.000	0.003	0.000

### Follow up results

Compared to before surgery, LAD, LVEDD and PA all declined after surgery and the difference had statistical significance; but LVEF had no obvious changes. LAD recovered faster than left ventricular diameter and main pulmonary artery diameter. One year later, LAD recovered to the normal level, but left ventricular diameter and main pulmonary artery diameter remained abnormal (Table-II).

**Table-II:** Comparison of color ultrasonography results before surgery, after surgery and in follow up (mean±SD).

*TTE*	*Before*	*24 ~ 72 h*	*One month*	*Three months*	*Six months*	*One year*
LAD (mm)	44.01±10.22	39.56±7.42^#^	37.43±8.12^#^	34.92±8.93^#^	37.31±10.72^*^	32.52±5.01^#^
LVEDD(mm)	65.37±13.86	61.93±10.68^*^	57.03±10.11^#^	54.42±9.71^#^	53.13±14.36^#^	49.86±7.94^#^
PA (mm)	40.72±8.76	35.00±7.36^#^	31.67±4.47^#^	29.93±4.48^#^	31.06±8.92^#^	28.00±3.87^#^
LVEF (%)	60.82±8.50	60.82±8.63	60.84±9.02	60.97±8.67	60.94±9.03	61.11±9.32

Note:

* means p<0.05 and

# means p<0.01 compared to before surgery.

TTE: Transesophageal echocardiography.

### Complications

One patients developed descending aortic stenosis and five patients showed chest pain. But the complications were mild and could be tolerated; afterwards, the symptoms gradually relieved. One patient was observed with trivial residual shunt but without hemolysis during follow up.

## DISCUSSION

Patent ductus arteriosus is a commonly seen congenital heart disease. Limited by economical and medical conditions in China, many patients suffering from patent ductus arteriosus go to hospital only when the symptoms are quite serious, but at that time, severe pulmonary hypertension has been induced. The measures for treating patent ductus arteriosus of patients with composite congenital heart disease are as follow. For young patients with patent ductus arteriosus but without pulmonary arterial hypertension, ligation or transfixion can be directly performed through median approach before CPB.^11^,^12^ Repairing patent ductus arteriosus with patch after heart blocking is also a frequently used therapy for treating large patent ductus arteriosus in combination with pulmonary arterial hypertension, but it seems to be a challenge for cardiac surgeon. That is because, high-pressure blood flow from main artery makes exposure difficult and severe outcomes such as perfusion lung and severe damage on blood components may be induced.^13^,^14^ To repair patent ductus arteriosus, hypothermia and flow perfusion and even suspension of circulation are usually adopted; however, the incidence of extracorporeal circulation associated complications becomes higher in such a condition. Hybrid surgery is also extensively used. Doctors perform occlusion therapy after establishment of CPB and then cardiac surgeons treat the other heart malformations.^15^ This technique is suitable for patients with small arterial diameter but without pulmonary arterial hypertension, but it also has defects such as X-ray radiation, contrast agent associated complication and high sensitivity to age and weight of patients. For patients with large arterial diameter and pulmonary arterial hypertension, interventional closure is not a good choice as closure device is easy to fall off.^16^

Our department innovatively applied the therapy combining pulmonary artery closure and patch technique for treating congenital heart disease combined with large patent ductus arteriosus, which combines the advantages of minimal traumatic occlusion and conventional extracorporeal circulation direct-view inner heart operation.^17^,^18^ The therapy greatly lowers surgical difficulty, shortens surgical time and also lowers surgical risks and the incidence of intraoperative and postoperative complications. Compared to previous surgical method, the therapy has the following advantages. First, it greatly lowers surgical difficulty and saves surgical time. Treatment of thirty-six cases was all fulfilled within 10 minutes in this study and doctors with initial experience of transthoracic minimal traumatic occlusion are capable of performing the surgery. In the whole surgical process, procures link up naturally. Besides, the selection of the type of closure device is relatively loose. Replacement of closure device is unnecessary even when there is a little residual shunt after closure (shunt bundle≤2 mm). Because most shunt has been blocked, which can provide a good view for patching? In addition, closure of large patent ductus arteriosus combined with patching lowers the incidence of recanalization, pseudoaneurysm and residual reflux. For patients with moderate or severe pulmonary arterial hypertension, closure device is easy to fall off. To avoid that situation, we tied a knot on pericardium using 3-Oprolene wire. What is more, surgical risks as well as the incidence of intraoperative and postoperative complications and CPB associated complications are low and X-ray radiation and contrast agent associated complications are avoided. The therapy is applicable for patients with any size of arterial diameter in combination with other heart malformations. But for patients with small arterial diameter and without pulmonary arterial hypertension, ligation may be more economical.

The size of closure device is usually 4 mm~6 mm larger than the diameter of arterial duct detected by transesophageal echocardiogaphy. In this study, 32 patients applied columnar and single-rivet closure device; 4 patients were treated with atrial septal defect closure device (20 mm). For window-type patent ductus arteriosus with diameter larger than 10 mm, many researchers advocate the application of atrial septal defect closure device.

### Limitations of the study

This is a single-center retrospective study and included small size of samples; therefore, the follow up results might be biased and prospective clinical studies with large sample size and multiple centers remain to be carried out. Besides, limited by invasiveness of the treatment and economical condition, follow up of most patients could only be possible through conventional physical examination, electrocardiograph and transthoracic and transesophageal echocardiography; and invasive cardiac catheterization were rarely used to confirm hemodynamic changes.

## CONCLUSION

Pulmonary artery closure in combination with patch technique is a simple operation which can shorten surgical time, lower the incidence of surgical complications, improve success rate and avoid X-ray radiation and contrast agent. Hence it is a new technology worth promotion.
